# Yeast Solutions and Hyperpolarization Enable Real-Time Observation of Metabolized Substrates Even at Natural Abundance

**DOI:** 10.1021/acs.analchem.4c02419

**Published:** 2024-10-15

**Authors:** Josh P. Peters, Charbel Assaf, Farhad Haj Mohamad, Eric Beitz, Sanjay Tiwari, Konrad Aden, Jan-Bernd Hövener, Andrey N. Pravdivtsev

**Affiliations:** † Section Biomedical Imaging, Molecular Imaging North Competence Center (MOIN CC), Department of Radiology and Neuroradiology, University Medical Center Kiel, Kiel University, Am Botanischen Garten 18, Kiel 24118, Germany; ‡ Pharmaceutical Institute, CAU Kiel, Gutenbergstr. 76, Kiel 24118, Germany; § Institute of Clinical Molecular Biology, Kiel University, Gut Rosalind-Franklin-Straße 12, Kiel 24105, Germany; ∥ Department of Internal Medicine I, University Medical Center Kiel, Arnold-Heller-Straße 3, Kiel 24105, Germany

## Abstract

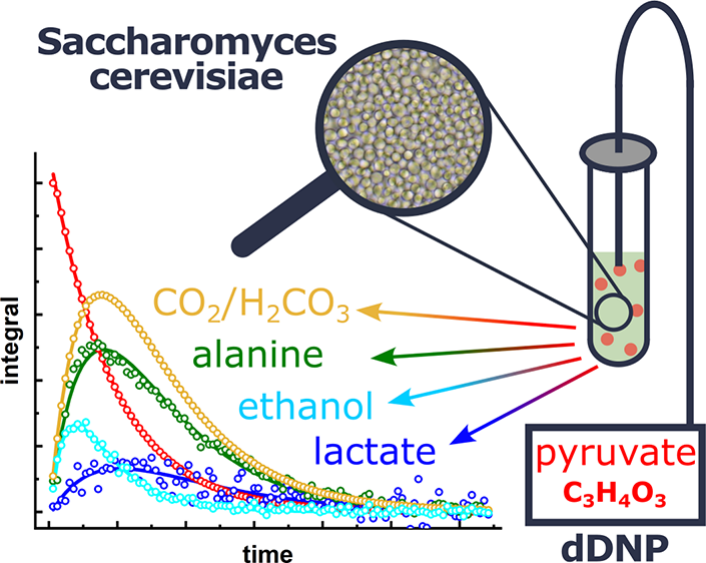

Metabolic changes in an organism often occur much earlier than macroscopic manifestations of disease, such as invasive tumors. Therefore, noninvasive tools to monitor metabolism are fundamental as they provide insights into in vivo biochemistry. NMR represents one of the gold standards for such insights by observing metabolites. Using nuclear spin hyperpolarization greatly increases the NMR sensitivity, enabling μmol/L sensitivity with a time resolution of about one second. However, a metabolic phantom with reproducible, rapid, and human-like metabolism is needed to progress research in this area. Using baker’s yeast as a convenient metabolic factory, we demonstrated in a single study that yeast cells provide a robust and rapidly metabolizing phantom for pyruvate and fumarate, including substrates with a natural abundance of ^13^C: we observed the production of ethanol, carbon dioxide, bicarbonate, lactate, alanine from pyruvate, malate, and oxaloacetate from fumarate. For observation, we hyperpolarized pyruvate and fumarate via the dissolution dynamic nuclear polarization (dDNP) technique to about 30% ^13^C polarization that is equivalent to 360,000 signal enhancement at 1 T and 310 K. Major metabolic pathways were observed using tracers at a natural abundance of ^13^C, demonstrating that isotope labeling is not always essential in vitro. Enriched [1-^13^C]pyruvate revealed minor lactate production, presumably via the D-lactate dehydrogenase (DLD) enzyme pathway, demonstrating the sensitivity gain using a dense yeast solution. We foresee that yeast as a metabolic factory can find application as an abundant MRI phantom standard to calibrate and optimize molecular MRI protocols. Our study highlights the potential of using hyperpolarization to probe the metabolism of yeast and other microorganisms even with naturally abundant substrates, offering valuable insights into their response to various stimuli such as drugs, treatment, nourishment, and genetic modification, thereby advancing drug development and our understanding of biochemical processes.

## Introduction

NMR is a powerful tool for studying metabolism due to its noninvasive, qualitative, and quantitative properties.^1^ Using hyperpolarized metabolites has enabled in vivo real-time metabolic imaging with great perspectives in medicine^2−4^ and analytical chemistry.^5^ Dissolution dynamic nuclear polarization^6^ is the leading technique for hyperpolarizing ^13^C-labeled pyruvate (and many other molecules) for subsequent molecular imaging and spectroscopy. High-resolution NMR with hyperpolarized substrates was used for studying plant extracts,^7^ metabolism in living cells,^8−12^ and organoids,^13^ revealing elusive intermediates and verifying mechanisms of chemical conversion quantitatively^14,15^ using ^13^C-labeled compound or with natural abundance (n.a.).^16^

Using hyperpolarized substrates, however, is no trivial task as its preparation and application consist of many steps: hyperpolarization, delivery, administration, and detection. The in vivo experimental trials offer the most realistic conditions, but they are also technically demanding and ethically difficult, especially if a large amount is needed. However, many tests involving hyperpolarized tracers and their metabolic conversions are necessary to optimize the experimental parameters.

Thus, to optimize observation procedures for hyperpolarized in vitro metabolic imaging or to demonstrate metabolism, enzymatic extracts,^17,18^ living,^18,19^ or lysed cells^20^ are typically employed. These approaches, however, come with some caveats, too. The enzymes are expensive, delicate to handle (e.g., temperature dependence, cosubstrate, pH), and degrade easily. Mammalian cells are similar and require special equipment that is not always compatible with, or available at, medical research centers (e.g., protective atmosphere, temperature control).

An alternative and simple approach is a chemically induced rapid chemical reaction of the hyperpolarized tracers exemplified by the decomposition of pyruvate by H_2_O_2_.^21^ While this approach is robust, cost-effective, and simple, it does not mimic the situation in vivo, as pointed out by the authors.

Conversely, yeasts are relatively robust, and their real-time metabolism has been demonstrated using hyperpolarized substrates.^15,22^ Yeasts and mammals, as both are eukaryotic organisms, share numerous biological functionalities, including similarities in cell cycle, metabolism, and other fundamental cellular pathways.^23−25^ Together with their rapid growth, their similar biological processes have elevated yeasts to be desirable model organisms for studying and understanding essential mechanisms found in higher eukaryotes, including humans.^26,27^ In addition, yeast gained relevance as a common platform for gene intervention.^28^

Here, we describe our approach using yeast cells as a biological surrogate system for hyperpolarized NMR and MRI. We introduce a straightforward way to prepare model solutions using food-grade dry yeast for optimization of hyperpolarization spectroscopy and imaging with ^13^C labeled [1-^13^C]pyruvate, pyruvate at n.a. of ^13^C (i.e., simultaneous co-hyperpolarization of [1-^13^C] and [2-^13^C]pyruvate), ^13^C labeled [1,4-^13^C_2_]fumarate and fumarate at n.a. of ^13^C. The pyruvate and fumarate at n.a. were enough to observe the most relevant metabolic steps, while labeled pyruvate led to the first observation of the D-lactate dehydrogenase (DLD) enzyme pathway^29^ using hyperpolarization. The observed kinetics were modeled using an open-source code for multiproduct metabolic reactions.

The work focused on establishing the protocol and measuring high-resolution spectral kinetics of yeasts-induced enzymatic reactions. The reaction rate can be adjusted as necessary because of the yeast availability, high achievable cell density, and observed linear conversion rate dependence on the yeast cell amount. All aspects of yeast work together to help develop novel metabolic spectroscopy and imaging techniques.

## Experimental Methods

### Hyperpolarization

All dDNP experiments were performed using a cryogen-free dDNP system (SpinAligner, Polarize) at ∼1.4 K and 6.7 T as detailed before.^30^ A microwave (MW) frequency between 187.045 and 187.105 GHz with 25 mW power was used for the polarization. For each dDNP experiment, 20–28 μL of the sample were taken from the stock, filled into the sample cup, and lowered into the MW cavity. DNP was initiated, and the buildup in the solid state was monitored every 2 to 5 min with a ^13^C RF pulse of 1°. After dissolution with 3.8–4 mL of superheated (∼200 °C, 11 bar) dissolution medium, the sample was transferred to an NMR system and detected 15 to 40 s later.

The pyruvate stock sample was prepared by mixing about 25 mg of trityl radical (AH111501, Polarize) and 640 mg of pyruvate (107360, Sigma-Aldrich) or [1-^13^C]pyruvate (677175, Sigma-Aldrich). This resulted in 31 mM trityl and 14 M pyruvate concentrations in the stock sample.

The fumarate stock sample was prepared by mixing about 21 mg of trityl radical , and 290 mg of [1,4-^13^C_2_]fumarate (755915, Sigma-Aldrich) dissolved in 600 mg of DMSO containing Gadolinium (Gadovist, Bayer). This resulted in 17 mM trityl, 3.2 M fumarate, and 0.6 mM Gadolinium concentrations in the stock sample. The sample composition with n.a. fumarate (47910, Sigma-Aldrich) consisted of 14.3 mg of trityl radical, 106 mg of fumarate, and 300 mg of DMSO. This resulted in 24 mM trityl, 2.5 M fumarate, and 0.6 mM Gadolinium.

After dissolution and before mixing with yeast, the sample concentration was around 90 mM (25 mg sample, and 3.8 mL dissolution volume) for both labeled and unlabeled pyruvate and 27 mM (32 mg sample, and 3.8 mL dissolution volume) for labeled and 32 mM (49 mg sample, and 3.7 mL dissolution volume) for unlabeled fumarate.

### Yeast Preparation

Seven grams of commercial dry yeast were dissolved in water combined with 0 or 1.2 g (0.2 M) of KH_2_PO_4_ buffer, yielding a total of 44 mL inside a 50 mL falcon tube. The tube was placed in a 305 K water bath and shaken every 2 min to aerate the solution and release CO_2_. An NMR tube was placed in the bath as well. After 10 min, the falcon was shaken again, and the desired amount of yeast was pipetted into the preheated NMR tube. The typical amount of yeast solution was 300 μL. The NMR tube was placed inside the NMR bore at 310 K. The cap of the tube was punched, and a thin tubing (ETFE 1/32″ x 0.25 mm, JR-T-084-M1.5, Vici Jour) was placed inside, about 2 mm below the surface of the yeast solution. The dissolution medium of the dDNP was heated subsequently. When the hyperpolarization solution was prepared, the NMR experiment started, and 300 μL of hyperpolarized and dissolved tracer was typically injected into the NMR. A similar forceful injection by hand was tested before.^11^ The dilution of the hyperpolarized medium in yeast solution resulted in about 45 mM concentration of pyruvate and 20 mM of fumarate.

### Microscopy Imaging

Yeast samples with dilutions of 10, 100, and 1000 times were placed in 8-well chambered cover glass (Nunc Lab-Tek Chambered Cover Glass, Catalog number: 155383, Thermo Scientific) and imaged with the Thunder imager 3D assay (Leica Microsystems GmbH) based on a Leica DMi8 microscope with 20x objective lens (HC PL APO 20*x*/0.80 dry) and Leica MC190 HD Microscope Camera. The produced images were processed using Leica LAS X software.

### Cell Counting

The yeast solution was diluted 100-fold and mixed with 0.4% trypan blue solution in a 1:1 ratio to distinguish viable and nonviable cells, resulting in a final dilution of 200. Subsequently, this mixture was loaded into a Neubauer counting chamber, and yeast cells were counted under a 40x objective lens of Leica DM IL microscope (Leica Microsystems GmbH) within four representative small squares located at the center of the grid (16 squares total with a volume of 0.1 mm^3^). The cell concentration was consequently determined as

Yeast cells/1 mL = Counted cells × Dilution × 10^4^

The standard deviation of three cell count measurements was determined to be 3.4%.

### NMR Experiments

^13^C MR signals were acquired in parallel using a 1 T ^13^C benchtop NMR (SpinSolve Carbon, Magritek) and a 9.4 T wide bore NMR with a narrow bore 5 mm BBFO probe (WB400, Avance NEO, Bruker). The transfer time to the 1 T system was *t*^1T^=(22.2 ± 4.1) s, and the transfer time to the 9.4 T system was *t*^9.4T^=(27.0 ± 3.6) s.

At 1 T, hyperpolarized spectra were acquired every 6 s with a 5° excitation angle (α) and a receiver gain of 31 or 70 dB for labeled or n.a. substrates, respectively. Thermally polarized, ^13^C enriched samples were averaged over 1800 to 3600 scans with α ∼ 90°, repetition time (TR = 2 s), and doped with the addition of 4 μL gadolinium (Gadovist, Bayer).

At 9.4 T, hyperpolarized spectra were acquired every 3 s with α ∼ 5°, ^1^H decoupling, and a receiver gain of 10 or 101 for labeled or n.a. substrates, respectively.

Measured polarization (*P*^1T^) and  on 1 T spectrometer with a transfer time *t*^1T^ was used to estimate the polarization in the yeast solution measured at 9.4 T in parallel with the transfer time of *t*^9.4T^. This was estimated using  with the following equation . Polarization was not measured inside the 9.4 T due to the ongoing strong consumption of substrates by the yeast, which would lead to overestimation of the polarization level. This estimate is sufficiently accurate, considering the average difference in transfer times *t*^1T^*- t*^9.4T^= 4.8 s and a relatively long relaxation time  s for [1-^13^C]pyruvate, (72.7 ± 1.3) s for [2-^13^C]pyruvate and (55.2 ± 3.4) s for [1,4-^13^C_2_]fumarate.

The polarization *P*^1*T*^ was (37.5 ± 6.1)% for labeled pyruvate, corresponding estimated *P*^9.4*T*^ was (35.4 ± 6.3)%. The sensitivity at 1 T was too low to quantify the polarization of pyruvate at natural abundance. Similar polarization levels were estimated by comparing the signal intensities of hyperpolarized n.a. pyruvate with the labeled pyruvate and adjusting for the ^13^C n.a. concentration. The polarization *P*^1*T*^ of fumarate was (28.8 ± 5.2)% and estimated *P*^9.4*T*^ was (30.8 ± 5.0)%.

### Analysis of NMR Data

*Signal processing and quantification:* An exponential appodization of 1 Hz was applied for the kinetic spectra and 3 Hz for polarization analysis. A third-order polynomial baseline correction was applied for each peak. Phase correction was applied in all cases. Integrals were used to quantify the signals (MNova, 14.2.2, Mestrelab Research S.L.).

*Quantification of polarization:* The signal of the first hyperpolarized spectrum at 1 T was used to calculate the polarization with respect to the thermally polarized spectrum at 1 T. A dual exponential function was fitted for *T*_1_ calculation. In yeast experiments, only the 1 T data was used to calculate the polarization because the yeast constantly consumed the substrate inside the 9.4 T NMR, while no yeast was used at 1 T.

Polarization from the yeast experiments was not calculated for n.a. substrates because the sensitivity at 1 T was too low. Instead, the polarization of n.a. substrates was measured at 9.4 T in the additional experiment without yeast solution.

The integrals of each substrate and up to four products were saved in a.csv file for further kinetics analysis. The detailed step-by-step guide to the analysis is available in SI.

### Analysis Guide for Yeast

A Python program was written to fit the first-order kinetics of all metabolites regarding the substrate. This was done using the Broyden–Fletcher–Goldfarb–Shanno algorithm to minimize the system of ordinary differential equations (ODEs) for the substrate and up to four products. The program is detailed in SI, and the corresponding Python script is available as supplementary. This leads to more accurate *T*_1,real_ for each metabolite, because the substrate *T*_1,real_ can be calculated more precisely by taking into account all metabolic pathways at once. As we do not include the cell uptake rates, the fitted exchange rates are a superposition of uptake and metabolism, if applicable.

If certain metabolites were not observed, its *k* was set to 0 inside the program. In some cases, the fit was improved by altering the penalty for residuals in the fit for the metabolites. The fitted variables were saved, and the fitted data was plotted for visual quality control.

## Results

### Preparation and Metabolic Reactor

Typically, 7 g of dried baker’s yeasts (*Saccharomyces cerevisiae*, SC) was dissolved in either deionized water (DI) or 0.2 M KH_2_PO_4_ buffered water to yield a total volume of 44 mL. The buffer was chosen due to its buffer range at neutral pH, the solution’s osmolarity, and its suggestion and use in literature.^31^ The solution was incubated in a water bath at 305 K for 10 min, yielding a 1.7 × 109 cells/mL density.

**Figure 1:**
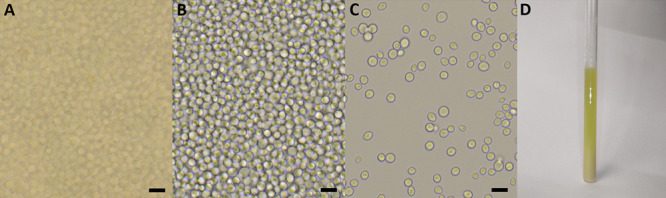
Microscopic images of yeast diluted 10 (A), 100 (B), and 1000 (C) times, and a photograph of the 5 mm NMR tube after the hyperpolarization experiment containing the yeast suspension in the former hyperpolarized solution (D). The size of the microscopic images (A-C) was 108.5 μm by 123.5 μm. Based on the cell counting by a hemocytometer, the concentration of a standard yeast solution was 1.7 × 10^9^ cells/mL with typically 0.51 × 10^9^ cells in the NMR tube (300 μL of stock yeast sample). The yeast cells were dissolved in deionized water; most cells appeared intact. Scale bar = 10 μm. For hyperpolarized NMR, we added 300 μL of hyperpolarized solution to the same volume of yeast suspension (2-fold dilution, D).

To commence hyperpolarization experiments, 300 μL of the yeast suspension equivalent to 0.51 × 10^9^ cells was withdrawn from the stock solution and filled into a 5 mm NMR tube, preheated to 305 K (Figure 1D).

A capillary (ETFE 1/32″ x 0.25 mm) was placed inside the NMR tube to inject the hyperpolarized media. We found that putting the capillary outlet 1–2 mm below the surface of the suspension was optimal for injecting the hyperpolarized solution to avoid the formation of bubbles; placing the capillary at the bottom of the tube resulted in trapped bubbles, poor magnetic field homogeneity, and poor mixing.

Next, the NMR tube with capillary was placed inside the NMR with temperature control at 310 K, and 300 μL of the hyperpolarized solution was injected shortly after. Before the experiments, the NMR system was calibrated to a similar 600 μL sample.

### Hyperpolarization of ^13^C Labeled [1-^13^C]pyruvate and Metabolism

Pyruvate, 99% ^13^C-enriched in the C1 position [1-^13^C]pyruvate and with an additional 1.1% natural abundance in the C2 position, [1,2-^13^C_2_]pyruvate, was polarized by dissolution DNP as previously described.^30^ About (24.8 ± 2.2) s after dissolution with a polarization of ≈ (33.5 ± 7.0)%, 300 μL of DNP solution was injected into the yeast bioreactor (ca. 0.51 × 10^9^ cells), and 5° excitation with subsequent NMR acquisition was applied. A strong signal of hyperpolarized pyruvate and its downstream metabolites was observed routinely.

**Figure 2:**
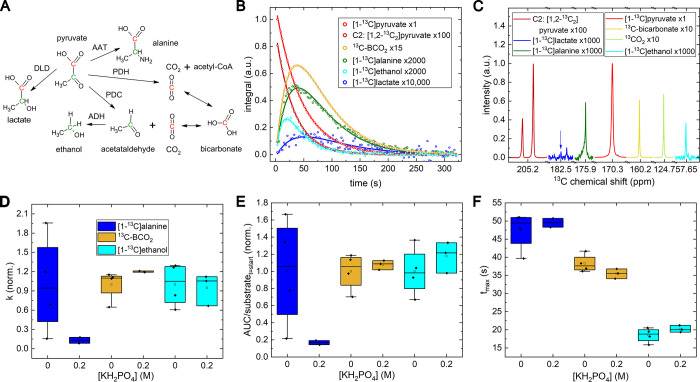
Overview of pyruvate metabolism in yeast cells using hyperpolarized and ^13^C labeled [1-^13^C]pyruvate. (A) Metabolic fate of the C1 and C2 carbons of pyruvate: The carbon in C1 position (red) is found in alanine, CO_2_, and bicarbonate, the C2 carbon (green) becomes part of acetaldehyde and ethanol. We use 99% ^13^C enriched C1 of [1-^13^C]pyruvate, and 1.1% ^13^C natural abundance C2 of [1,2-^13^C_2_]pyruvate. (B) Integrated NMR signals (indicators) and fit (lines) measured after adding hyperpolarized pyruvate solution to yeast as a function of time. (C) ^13^C NMR spectra of each resonance at their individual time point of maximum (*t*_max_): 25 s after dissolution for [1-^13^C]pyruvate and n.a. C2 of [1,2-^13^C_2_]pyruvate, 75.2 s for [1-^13^C]lactate, 64.7 s for [1-^13^C]alanine, 63.3 s for [^13^C]BCO_2_, and 43.1 s for n.a. [1-^13^C]ethanol. A strong difference in signal and SNR can be observed between the products: ethanol, alanine, lactate, CO_2_ and bicarbonate. A first-order kinetics model fitted to the integrated signal intensities yielded the (D) conversion constants for pyruvate to lactate, *k*^pl^, alanine, ^kpa^, BCO_2_, *k*^pbc^, and ethanol, *k*^pe^. *k*^pa^ dropped dramatically if buffer was used, while *k*^pbc^ and *k*^pe^ were roughly constant. (E) The same model was used to calculate the area under the curve (*AUC*), normalized by division by the initial substrate signal at the metabolization start, and (F) the time to maximum signal (*t*_max_). In (D) and (E), the values were normalized to the unbuffered group’s mean value for each substrate. The absolute *k* values are in SI, Table S1. Polarization was (33.5 ± 7.0)% after (24.8 ± 2.2) s transfer, and pH in the NMR tube was 5.9 ± 0.2. DLD: D-lactate dehydrogenase, AAT: alanine aminotransferase, PDH: pyruvate dehydrogenase, PDC: pyruvate decarboxylase, ADH: alcohol dehydrogenase.

The strongest signal was observed in the C1 pathway of pyruvate conversion into bicarbonate (HCO_3_^–^) and CO_2_, reaching 1/10 of the maximum pyruvate signal. Less intense signals were observed for [1-^13^C]alanine, with a signal of about 1/2000, and [1-^13^C]lactate, with a signal close to the noise (Figure 2).

The ratio between CO_2_ and bicarbonate in the buffered solution was ≈0.87, indicating a pH of 5.96 according to the H. Henselbach equation,^32^ and close to the buffered value of 5.9 ± 0.2 (p*K*_a_ of bicarbonate is about 6.1).^33^ Because of the rapid exchange of CO_2_ and bicarbonate, we will report their combined signal from here on as BCO_2_.

Interestingly, and to our knowledge, for the first time, we were able to observe the C2 pathway at natural abundance, too. Hyperpolarized signal of [1-^13^C]ethanol was clearly observed, although the intermediate acetaldehyde was not (Figure 2A).

We designed a script that can simultaneously fit all observed kinetics to analyze the observed multiparametric metabolic transformations (the Python script is available in Supporting Information - SI). We used ODEs to fit observed metabolic kinetics to the signal intensities using the Broyden-Fletcher-Goldfarb-Shanno algorithm to obtain the conversion rate constants of pyruvate to lactate, *k*^pl^, alanine *k*^al^, BCO_2_, *k*^pbc^, and ethanol, *k*^pe^, the areas under curve (*AUCs*), and the time to the maximum signal (*t*_max_).

Note that the C1 ^13^C signal of ethanol originates from the polarization of C2 of pyruvate. Moreover, in our case of ^13^C labeled [1-^13^C]pyruvate, this signal originates from molecules with ^13^C in both C1 and C2 positions (double label). The concentration of this molecule, [1,2-^13^C_2_]pyruvate, is about 1.1% of the overall 95 mM pyruvate concentration in the hyperpolarized solution before mixing with the yeast—the additional carbon–carbon interactions in the double-label molecule cause additional line splitting and relaxation.

### Co-Hyperpolarization of [1-^13^C]pyruvate and [2-^13^C]pyruvate at ^13^C Natural Abundance and Metabolism

Since the metabolism of [1-^13^C] enriched pyruvate in yeast was so pronounced due to an about 100 times higher cell density compared to typical cell experiments, we decided to test yeast using pyruvate at natural abundance.

**Figure 3:**
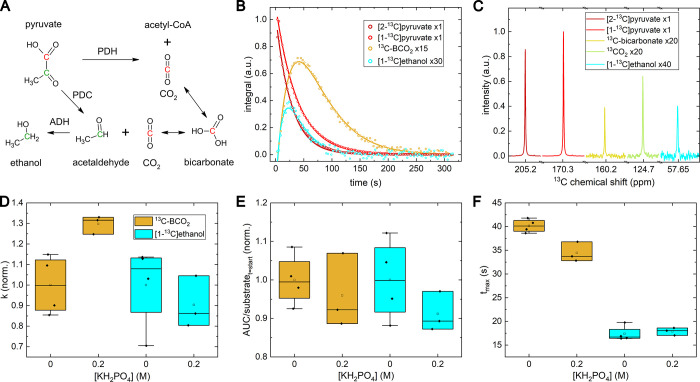
Overview of pyruvate metabolism in yeast cells using cohyperpolarized [1-^13^C] and [2-^13^C]pyruvate at an n.a. of ^13^C. (A) Metabolic fate of the C1 and C2 carbons of pyruvate: The carbon in C1 position (red) is found in CO_2_ and bicarbonate, the C2 carbon (green) becomes part of acetaldehyde and ethanol. (B) Integrated NMR signals (indicators) and fit (lines) measured after adding hyperpolarized pyruvate to yeast as a function of time. (C) ^13^C NMR spectra of each resonance at their individual time point of maximum (*t*_max_): 26.2 s after dissolution [1-^13^C]pyruvate and [2-^13^C]pyruvate, 63.0 s for BCO_2_, and 44.8 s for [1-^13^C]ethanol. Only a slight difference in signal and SNR can be observed between CO_2_, bicarbonate, and ethanol. No alanine or lactate was observed in contrast to the ^13^C labeled pyruvate experiments (Figure 2). A first-order kinetics model fitted to the integrated signal intensities yielded the (D) conversion constants for pyruvate to lactate, *k*^pl^, alanine, *k*^pa^, BCO_2_, *k*^pbc^, and ethanol, *k*^pe^. *k*^pa^ dropped dramatically if buffer was used, while *k*^pbc^ and *k*^pe^ were roughly constant. (E) The same model was used to calculate the area under the curve (*AUC*), normalized by division by the initial substrate signal at the metabolization start, and (F) the time to maximum signal (*t*_max_). In (D) and (E), the values were normalized to the unbuffered group’s mean value for each substrate. The absolute *k* values are in SI, Table S2. Solid state polarization was about 50% and the transfer took (28.0 ± 3.6) s transfer, pH in the NMR tube was 6.2 ± 0.2. PDH: pyruvate dehydrogenase, PDC: pyruvate decarboxylase, ADH: alcohol dehydrogenase.

At n.a. of ^13^C, the C1 and C2 carbons of pyruvate are almost equally hyperpolarized and have similar integrals about 20 s after dissolution (Figure 3). The receiver gain of the NMR was maximized (10 times higher than in experiments with labeled ^13^C) without receiver ADC saturation (overflow) due to an about 100 times smaller signal from the n.a. concentrated ^13^C. On some devices, this increase in receiver gain may yield a boost in sensitivity, which explains our initiative to change the receiver gain value. However, after careful analysis of the actual performance of our NMR, we have not noticed such an improvement in changing the receiver gain value from 10 to 101.^34^

In this experiment, we see that BCO_2_ derived from [1-^13^C]pyruvate has about twice the signal of [1-^13^C]ethanol derived from [2-^13^C]pyruvate; however, their rate constants appear pretty similar.

The use of n.a. pyruvate was found to be beneficial as it allowed simultaneous observation of both carbons, following their fate without additional ^13^C–^13^C splitting (see C2 signal in Figure 2C), which leads to an increase in line separation and spectroscopic sensitivity. Using yeast assay with a 100-times higher cell density than in typical experiments with human or mouse cells compensates for the signal loss from using n.a. ^13^C compared to^13^C enriched tracers.

Similar to before, when we used labeled [1-^13^C]pyruvate, we saw increased BCO_2_ production in the buffered solution compared to deionized water (Figures 2D, 3D). Ethanol was not significantly affected.

### Fumarate Metabolism

**Figure 4:**
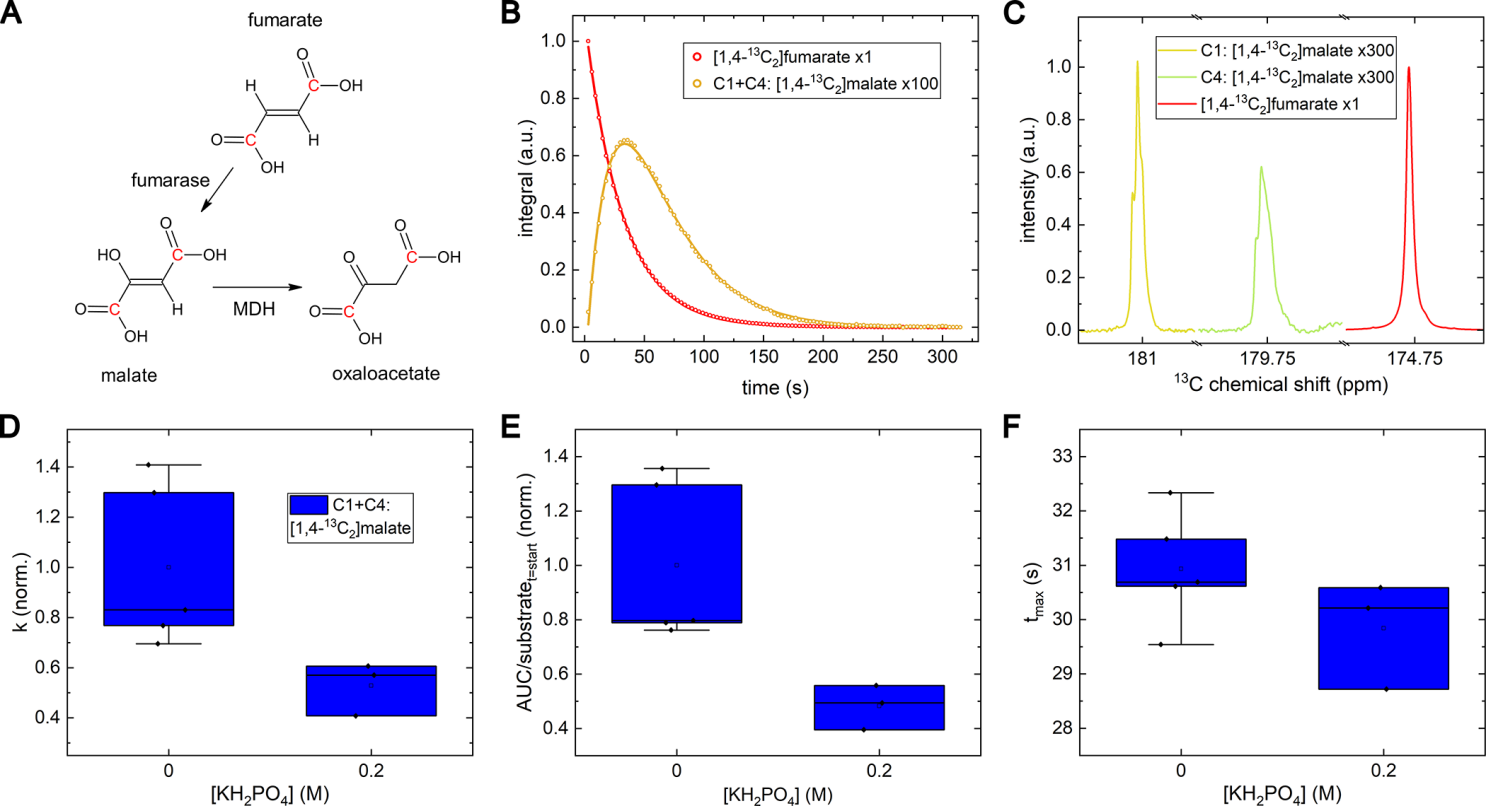
Overview of fumarate metabolism in yeast cells using hyperpolarized and ^13^C labeled [1,4-^13^C_2_]fumarate. (A) A chemical conversion diagram of fumarate with highlighted C1 and C4 carbons (red) into malate and oxaloacetate. (B) Integrated NMR signals (indicators) and fit (lines) were measured after adding hyperpolarized fumarate to yeast as a function of time, and (C) ^13^C NMR spectra acquired signals are shown for each resonance at their individual time point of maximum (*t*_max_): acquired 26 s after dissolution for [1,4-^13^C_2_]fumarate and 56.7 s for C1 and C4 of [1,4-^13^C_2_]-malate. Two peaks of malate were combined for kinetics analysis for a better sensitivity.). A first-order kinetics model fitted to the integrated signal intensities yielded the (D) conversion constants for fumarate to malate, *k*^fm^. (E) The same model was used to calculate the area under the curve (*AUC*), normalized by division by the initial substrate signal at the metabolization start, and (F) the *t*_max_. In (D) and (E), the values were normalized to the unbuffered group’s mean value for each substrate. A strong difference between the DI and buffered yeast was found for *k*^fm^ and the area under the curve but not for the *t*_max_. The absolute *k* values are in SI, Table S3. Polarization was (28.8 ± 5.2)% after (29 ± 4.4) s transfer, and pH in the NMR tube was 5.6 ± 0.3. MDH: malate dehydrogenase.

Fumarate is one of the most popular studied molecular imaging tracers, as it allows necrosis diagnosis.^35−37^ In yeast, fumarate quickly converts to malate examplified using hyperpolarized ^13^C labeled [1,4-^13^C_2_]fumarate(Figure 4) and n.a. fumarate (Figure S1, SI), which is comparable to humans. Hence, yeast again presents a good platform for converting fumarate to malate, with the malate signal only about 100 times lower than fumarate. The concentration of malate at its *t*_max_ was close to 3% of the fumarate concentration.

An addition of KH_2_PO_4_ buffer decelerated the production of malate from fumarate.

Experimentally, we observed signals of oxaloacetate in some cases, with the signal being in the order of noise, indicating that the metabolism is present but too slow compared to the relaxation or it is proceeding into the Krebs cycle at a rapid rate.

### Sample Adjustments

**Figure 5:**
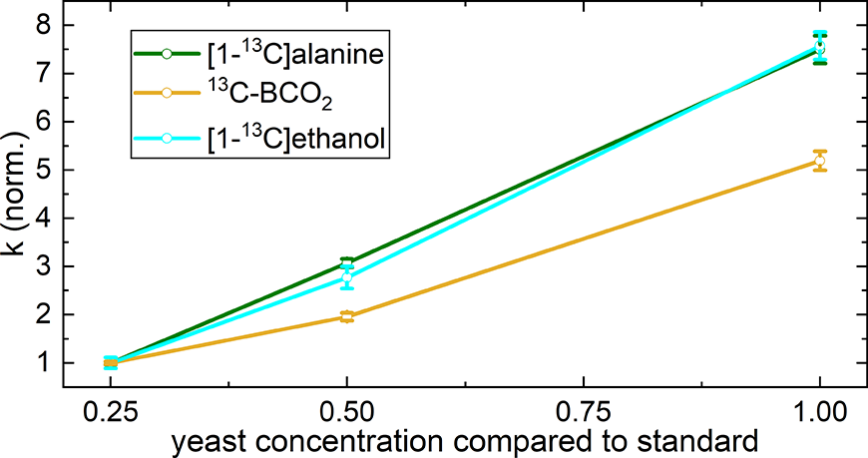
The fitted conversion exchange rate constants as a function of the yeast concentration. The concentration of yeast was altered by adding varying amounts of yeast concentrate, keeping the same amount of hyperpolarized pyruvate. The reduction of yeast volume was substituted with deionized water to keep the sample at the same total volume. A proportional increase in conversion rates with yeast concentration was observed for all products.

We carried out all previously described experiments using samples with 300 μL of yeast and an additional 300 μL of hyperpolarized dDNP solution. Here, we tested the effect of a varying amount of yeast relative to the hyperpolarized medium. The yeast concentrate was diluted in water at 300:0, 150:150, and 75:225 before the addition of a hyperpolarized medium. For the experiments that were conducted, the conversion rate changed proportionately with the concentration (Figure 5).

Second, we found that one can adjust the position of yeast or cells in the NMR tube using 0.5 mm glass beads.^38^ This was useful to position the yeast cells directly into the sensitive area of the NMR tube. The amount of beads was weighed before the experiment, and their total volume was calculated. To compensate, we reduced the liquid volume while keeping a constant ratio of yeast and hyperpolarized medium, resulting in the solution’s constant concentrations of pyruvate and yeast.

**Figure 6:**
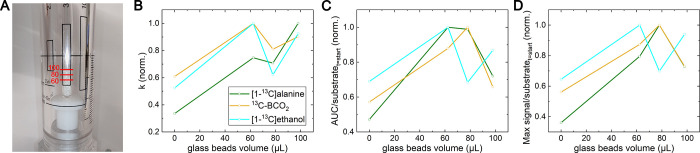
Metabolic data from pyruvate metabolism in yeast depending on the position of the yeast in the NMR tube. We added a varying volume of 0.5 mm glass beads to the bottom of the NMR tube to keep the yeast predominantly in the sensitive area of the NMR coil. (A) Photo of the NMR tube with the maximum tested amount of beads and indicated tested glass beads volumes. The fitting of the kinetics provided (B) the conversion rate constant *k* and (C) the area under the curve (*AUC*). (D) Observed maximum intensity of the products relative to the initial signal of the substrate. The polarization was close to constant in all experiments. An increase in product signals showed a benefit when adding the glass beads, though the lowest tested amount of glass beads (156 mg or ∼60 μL) seems to be enough. Polarization was (34.9 ± 2.4)% after (27.4 ± 1.6) s, and pH in the NMR tube was 6.3 ± 0.1. The weight of the beads was measured and then converted into volume using the typical glass density of 2500 kg/m^3^.

With this approach, we measured the signal of the metabolites and pyruvate as a function of the beads’ volume. Across four experiments, the quantified polarization of [1-^13^C]pyruvate at 9.4 T was (34.9 ± 2.4)%, and the average transfer time was (27.4 ± 1.6) s. We observed an increase in the apparent signal of the products, although the sensitive volume was partially covered with glass beads (Figure 6). We also found a stronger observable production of lactate while using glass beads; without them, it was only observed a single time.

We noticed that if yeast flows inside the area of the glass beads, they can produce so much CO_2_ that part of the beads will be lifted up inside the tube. To avoid this, one can fill the volume around glass beads with the buffer solution first, add a filter on top,^38^ or use Shigemi–like NMR tubes.

## Discussion

Our approach of leading the yeast into the NMR proved to be very simple and robust, and it should be easily repeatable and implementable by other NMR laboratories. More stable conditions, and thus more reproducible results, may be achieved through the use of portable cell reactors tailored to NMR.^38^ These reactors, however, increase the complexity of the experiment.

### General Considerations

The lifetime of the longitudinal magnetization *T*_1_ (and *T*_2_ also) depends on the magnetic field and the molecular environment. Previous studies found that the intracellular *T*_1_ relaxation rate of ^13^C tracers can be 2–4 times faster than extracellular relaxation.^39^ This difference may lead to an over- or underestimation of enzymatic conversion rates. Generally, quantifying conversion constants is a complex matter. The interplay of cellular uptake and actual conversion is often ignored, and only the “apparent” conversion rate is reported. However, these apparent or observed rates are still useful for biochemical analysis and medical diagnostics.

To distinguish intra and extracellular metabolites, one can change the extracellular pH and observe a shift in the resonance. However, this will also alter the uptake rate;^39^ instead, we aimed to carry out experiments close to physiological conditions.

### [1-^13^C]pyruvate Metabolism

Most of the exchange rates were in the order of 10^–6^ 1/s (alanine and lactate) to 10^–3^ 1/s (BCO_2_ and ethanol). Interestingly, almost 10% of the initial pyruvate concentration was reached for BCO_2_ at its *t*_max_ (∼40 s) in some cases, which indicates a very efficient uptake and metabolism of the pyruvate provided. The concentration of BCO_2_ was estimated by using the fitted rate constants and the initial pyruvate concentration in the solution.

The distribution of fitted exchange rate constants, *k*, (Figure 2D) to produce alanine, BCO_2_, and ethanol is very similar to the corresponding distribution of normalized AUCs (Figure 2E) that supports the common approach for using phenomenological AUCs instead of fitted rate constants.^40^

Adding 0.2 M of potassium salt (0.4 osmol/L), optimal for yeast,^31^ did not change the observed metabolism of BCO_2_ and ethanol much (Figure 2D,E,F). Notably, potassium salt acts as a buffer at pH 4.1–4.5, the optimal pH range for yeast. Thus, the finding that the BCO_2_ metabolism of yeast is not affected much by pH and osmolarity indicates that yeast cells have some resilience against environmental stress by these factors. This feature is advantageous in the scope of a hyperpolarization experiment as adding the hyperpolarized solution inevitably leads to environmental changes by slightly different pH or molecular density. Thus, adding compounds like the tracer and radical appears not to significantly impact the investigated system, which will lead to more reproducible results overall.

However, one can see that when the buffered solution was used, the conversion rate characteristics had a smaller deviation than the unbuffered solution.

We also found more substantial variability in metabolism for metabolites with lower conversion, such as alanine or lactate, indicating that such pathways are more prone to change or that the lower sensitivity induces unwanted fluctuations during the observation and analysis. This underlines the benefits of yeast’s strong metabolism by increasing the metabolite concentrations while providing human-like metabolic pathways.

The observation of lactate (Figure 2B,C) in our [1-^13^C]pyruvate experiments came as a surprise, as it was not reported before in SC using hyperpolarized tracers. This finding may be attributed to the large number of yeast cells investigated here, although the SNR was still low (∼2). However, due to the large number of data points, the standard deviation of the fit is rather good: *k*^pl^ is (6.82 ± 0.24) × 10^7^ 1/s in Figure 2.

Unlike ethanol in the fermentation process, SC does not express a human-like lactate dehydrogenase,^29^ so lactate is not readily produced from pyruvate. The only pathway from lactate to pyruvate is via the DLD enzyme, a zinc-flavoprotein. Apparently, the enzyme activity is affected by the presence of carboxylic acids such as pyruvate,^29^ which could contribute to the production of lactate in our case. Lactate was seen more frequently when glass beads moved yeast into the sensitive area of the NMR probe, and sensitivity increased during the measurement (glass beads experiments discussed below in *Sample adjustment*). Note that electroporation can increase metabolic rates a fewfold more.^41^

The observation of the fermentation pathway may be improved by using ^13^C labeled [2-^13^C]pyruvate or n.a. pyruvate where the double label is rare (1.1% have ^13^C in C1, same for C2, only 100 ppm have a double label in C1 and C2, and ≈97.8% have no ^13^C label). Therefore, using n.a. pyruvate effectively constitutes a co-hyperpolarization of [1-^13^C]pyruvate and [2-^13^C]pyruvate.

### Pyruvate Metabolism at Natural Abundance

Using substrates in natural abundance comes with an inherently lower sensitivity than labeled substrates. However, there are many ways to improve the sensitivity: One can utilize stronger magnets, cryoprobes, or ^13^C to^1^H polarization transfer with consequent^1^H observation,^12^ or use multiple samples to acquire several single spectra with maximum sensitivity at variable time points.^15^ Here, we aimed to demonstrate that using simple techniques and well-polarized precursors, the quantitative analysis and optimization of experimental techniques using n.a. substrates are possible in our strongly metabolizing model system. Key metabolic pathways can be tracked using the most basic experimental techniques and unlabeled compounds. Therefore, using n.a. substrates was found to be beneficial as it allows the observation of metabolic conversion to relevant products while being significantly cheaper than the labeled substrates.

We showed that BCO_2_ derived from [1-^13^C]pyruvate has about twice the signal of [1-^13^C]ethanol derived from [2-^13^C]pyruvate. This can be explained by a more rapid relaxation of ethanol compared to BCO_2_ (11.2 ± 1.4 s against 35.4 ± 4.5 s) and [2-^13^C]pyruvate compared to [1-^13^C]pyruvate (34.5 ± 2.6 s against 51.4 ± 2.4 s), as well as slightly faster conversion rates to BCO_2_ than to ethanol (of (2.92 ± 0.46) × 10^–3^ 1/s against (2.15 ± 0.35) × 10^–3^ 1/s)). Different polarization levels of the C1 and C2 pyruvate may also contribute to this effect.

BCO_2_ is produced from pyruvate in a one-step enzymatic reaction in two pathways through either pyruvate dehydrogenase (PDH) or pyruvate decarboxylase (PDC). On the other hand, ethanol is produced in only one pathway and in two steps: first, PDH converts pyruvate into acetaldehyde and BCO_2_, and then, with the help of alcohol dehydrogenase (ADH), it is converted into ethanol. So, for every ethanol, one CO_2_ is produced. It follows that the production of BCO_2_ should be the same or higher than ethanol production. The fact that the conversion rates of BCO_2_ and ethanol production are quite similar (Figures 2D, 3D) indicates that the alcoholic fermentation PDC/ADH pathway is dominating in contrast to the oxidative PDH pathway.

The addition of potassium salt accelerated the production of BCO_2_ and decelerated ethanol production (Figures 2D, 3D). Our measurements cannot give a conclusive explanation. The possible reasons are environmental stress (pH and osmolarity), which leads to alterations in the yeast’s metabolic behavior, increasing PDH activity (oxidative metabolism) and decreasing PDC/ADH.

Interestingly, we never saw acetaldehyde (a potent mutagen) as an intermediate of pyruvate to ethanol conversion. This is likely due to its rapid conversion to the end product within the cell and, hence, low steady-state concentration.^42^ Interestingly, different metabolic products^14,22^ and more intermediates can be observed under pH or redox stress, including increased accumulation of acetaldehyde.^11^ Using ^13^C–^1^H INEPT or ^13^C–^1^H cross-polarization^1^H observation schemes together with the use of [2-^13^C]pyruvate also increased sensitivity, and the acetaldehyde was observed;^12^ both factors (different observation scheme and labeled substrate) led to increased SNR compared to our experiment.

### [1,4-^13^C_2_]fumarate Metabolism

The fumarate uptake into cells is relatively slow. A rapid fumarate to malate conversion is typically observed in necrotic tissue, where the released fumarase from lysed cells facilitates the conversion in the extracellular space. Our observation of increased conversion in the unbuffered solution may be explained by the lysis of some yeast cells due to the osmotic stress in deionized water,^43^ unlike in the buffered solution.

However, the subsequent release of fumarase into the solution only increased the conversion rate *k*^fm^ by a factor of 1.9 (Figure 4), indicating that many of the cells stayed intact in both cases. The fact that strong metabolism was observed even in the buffered solution shows that uptake into the yeasts was sufficient to observe metabolism during the experiment. This partially results from some fumarate (p*K*_a_ 4.3)^44^ being protonated at the acidic yeast pH of 5.6 ± 0.3 and thus diffuses into the cells freely. This aspect highlights the benefits pH resilience of yeast, enabling the observation of carboxylic acid metabolism without the need for slow transporters.^39^

### Sample Optimization

Our observations with increasing yeast concentrations showed that although the yeast cells were relatively densely packed (Figure 1), their metabolism was unaffected. Hence, one can adjust the conversion rates as desired by changing the amount of yeast.

The higher conversion rates using glass beads result from a higher product concentration in the sensitive area of the coil and because the yeast is not settling at the bottom of the tube outside but rather on top of the beads and inside the sensitive area of the coil. This increases the apparent product concentration.

## Conclusion

Yeast cells were found to be a very convenient factory for converting pyruvate to CO_2_, bicarbonate, and ethanol, and fumarate to malate, which was demonstrated using hyperpolarized^13^C labeled and n.a. pyruvate and fumarate. Moreover, for the first time, we observed the production of lactate from pyruvate by the DLD pathway in yeast using hyperpolarized NMR.

Yeast suspension is straightforward to prepare at high concentrations before the experiment with any required cell density. The conversion rate is proportionally dependent on the yeast density and can be ancillary controlled by changing the ion density and pH. Using 0.5 billion cells, about 10% of 25 mM hyperpolarized pyruvate was converted into CO_2_ and bicarbonate within a minute.

Although most of the experiments shown here were carried out in standard NMR tubes, we showed that adding affordable glass beads improves the SNR and interaction between the cells and hyperpolarized medium even further.

Therefore, yeast cells can become a standard solution for comparing hyperpolarization and imaging performance due to their robustness, simplicity, and availability. This is especially true for the cases when novel hyperpolarized substrates at n.a. are used. We could standardize the conversion parameters using our multiparametric fitting program (see details in SI), which eases the comparability across data sets and reduces artifacts that influence the kinetic data sets.

Using pyruvate hyperpolarized to >50% in solid-state at a natural abundance of ^13^C, we show that isotope labeling is unnecessary even for a modest 9.4 T spectrometer with a room temperature probe. Using stronger magnets equipped with cryoprobes, observation of many hyperpolarized tracers will be possible as no isotope labeling is necessary, with the SNR being more than sufficient for analysis.

The measurement of hyperpolarized molecules, which takes only a few minutes, can save time on high-field NMR systems but also requires a lengthy preparation of hyperpolarization. However, more rapid production of hyperpolarization was recently demonstrated using parahydrogen,^19,45,46^ or multiprobe DNP systems,^47^ or rapid DNP generation.^7,48^ These methods will improve the throughput of metabolic experiments using hyperpolarization.

One can use many different stimulants to alter the yeast metabolism as desired.^22,49^ In addition, we expect that as yeast rapidly proliferates, one can use the technique developed here to test the changes in metabolism induced by gene modifications.^50^ This makes yeast a great platform for advancing hyperpolarization and, vice versa, makes hyperpolarization an excellent tool for yeast engineering.

## Data Availability

The necessary raw and analyzed data sets available on Zenodo [DOI: 10.5281/zenodo.13150701].

## Supporting Information Available

Details on spectral analysis and fitting of the metabolic kinetics, together with the used Python script (kinetics.py) (ZIP)
