# Supplementary material for: Yeast Solutions and Hyperpolarization Enable Real-Time Observation of Metabolized Substrates Even at Natural Abundance
Source: Anal Chem. 2024 Oct 15;96(43):17135–44. doi: 10.1021/acs.analchem.4c02419 (PMC11525923; doi:10.1021/acs.analchem.4c02419)
Supplement: Supplementary file 1 — ac4c02419_si_001.zip [file ac4c02419_si_001.zip › Supporting_information.pdf]

Supporting materials for

Yeast solutions and hyperpolarization enable real-time observation of metabolized substrates even at natural abundance.

Josh P. Peters[1], Charbel Assaf[1], Farhad Haj Mohamad[1], Eric Beitz[2], Sanjay Tiwari[1], Konrad Aden[3,4], Jan-Bernd Hövener[1*] and Andrey N. Pravdivtsev[1*]

AUTHOR ADDRESS

1. Section Biomedical Imaging, Molecular Imaging North Competence Center (MOIN CC), Department of Radiology and Neuroradiology, University Medical Center Kiel, Kiel University, Am Botanischen Garten 18, Kiel, 24118, Germany

2. Pharmaceutical Institute, CAU Kiel, Gutenbergstr. 76, Kiel, 24118, Germany

3. Institute of Clinical Molecular Biology, Kiel University, Gut Rosalind-Franklin-Straße 12, Kiel, 24105, Germany

4. Department of Internal Medicine I, University Medical Center Kiel, Arnold-Heller-Straße 3, Kiel, 24105, Germany

*Corresponding authors:

Prof. Jan-Bernd Hövener: jan.hoevener@rad.uni-kiel.de

Dr. Andrey N. Pravdivtsev: andrey.pravdivtsev@rad.uni-kiel.de

Contents

Metabolic data corresponding to figures S-2

Model for metabolic analysis software S-5

Protocol for the analysis of hyperpolarization spectroscopy S-8

Metabolic data corresponding to figures

Table S1: *Metabolic data of [1-^{13}C]pyruvate (PYR) and its metabolites. The rate constants with standard deviations are obtained using our multiparametric fitting of chemical exchange.*

1-13C-pyruvate results									
experiment	[KH2PO4] (M)	LAC k (1/s)	LAC k StDev (1/s)	ALA k (1/s)	ALA k StDev (1/s)	CO2 k (1/s)	CO2 k StDev (1/s)	Ethanol k (1/s)	Ethanol k StDev (1/s)
JAM229	0	no metab	no metab	9.47E-06	1.65E-07	3.00E-03	7.60E-05	2.31E-03	3.59E-05
JAM230	0	no metab	no metab	1.23E-06	7.00E-08	2.84E-03	1.69E-04	1.52E-03	6.66E-05
JAM231	0	6.82E-07	2.36E-08	1.55E-05	5.94E-07	2.88E-03	1.09E-04	2.36E-03	8.94E-05
JAM260	0	no metab	no metab	5.46E-06	5.15E-08	3.15E-03	4.58E-05	1.22E-03	3.43E-04
JAM233	0.2	no metab	no metab	no metab	no metab	3.11E-03	2.76E-04	2.04E-03	1.17E-04
JAM234	0.2	no metab	no metab	1.40E-06	8.20E-08	3.10E-03	1.90E-04	1.92E-03	1.40E-06
JAM239	0.2	no metab	no metab	6.67E-07	5.30E-08	1.68E-03	1.84E-05	1.11E-03	6.29E-05

Table S2: *Metabolic data of ^{13}C naturally abundant pyruvate (PYR) and its metabolites. The rate constants with standard deviations are obtained using our multiparametric fitting of chemical exchange.*

n.a. pyruvate results					
experiment	[KH2PO4] (M)	CO2 k (1/s)	CO2 k StDev (1/s)	Ethanol k (1/s)	Ethanol k StDev (1/s)
NAPYR1	0	2.83E-03	9.41E-05	2.55E-03	9.48E-05
NAPYR2	0	2.21E-03	5.39E-05	1.58E-03	1.20E-04
NAPYR3	0	2.97E-03	6.97E-05	2.32E-03	1.92E-04
NAPYR8	0	2.33E-03	4.58E-05	2.53E-03	7.84E-05
NAPYR4	0.2	3.23E-03	6.15E-05	1.81E-03	1.27E-04
NAPYR5	0.2	3.44E-03	5.37E-05	1.94E-03	1.06E-04
NAPYR6	0.2	3.40E-03	7.57E-05	2.35E-03	1.68E-04

Table S3: *Metabolic data of [1,4-$^{13}C_2$]fumarate (FUM) and its metabolites. The rate constants with standard deviations are obtained using our multiparametric fitting of chemical exchange.*

1,4-13C2-fumarate results			
**experiment**	**[KH2PO4] (M)**	**Malate k (1/s)**	**Malate k StDev (1/s)**
FUM15	0	9.00E-04	8.66E-06
FUM20	0	5.33E-04	3.94E-06
FUM22	0	4.82E-04	3.25E-06
FUM28	0	5.76E-04	4.73E-06
FUM29	0	9.77E-04	5.17E-06
FUM25	0.2	3.96E-04	3.24E-06
FUM26	0.2	4.21E-04	3.73E-06
FUM27	0.2	2.83E-04	2.77E-06

Metabolism of fumarate at natural abundance

A sample of fumarate at natural abundance was prepared for dDNP: 106 mg n.a. fumarate (47910, Sigma-Aldrich), 14.3 mg trityl radical (AH111501, Polarize) dissolved in 300 mg DMSO containing gadolinium (Gadovist, Bayer). This resulted in 2.5 M fumarate, 24 mM trityl and 0.6 mM gadolinium. 49 mg of the stock sample were polarized and dissolved in a 3.7 mL dissolution medium to achieve a 32 mM fumarate solution at a pH of 5.9. The polarization achieved was not estimated because the thermal signal was too low.

The signal achieved was about 100x lower compared to labeled fumarate, due to the difference in abundance. Despite the lower signal, a signal of 1- and 4-^{13}C-malate was observed using a receiver gain of 18 and a flipangle of 10° with repetition time of 4 s. Due to the low SNR of malate, the signal-to-noise ratio (SNR) was increased by summing up 4 spectra (**Figure S1**). Using this approach the time resolution was not enough to accurately fit the curve provided by the measured and summed points. However using more yeast, a higher fumarate concentration and/or a spectrometer with a cryo-probe or higher field-strength than 9.4 T should enable a sufficient analysis.

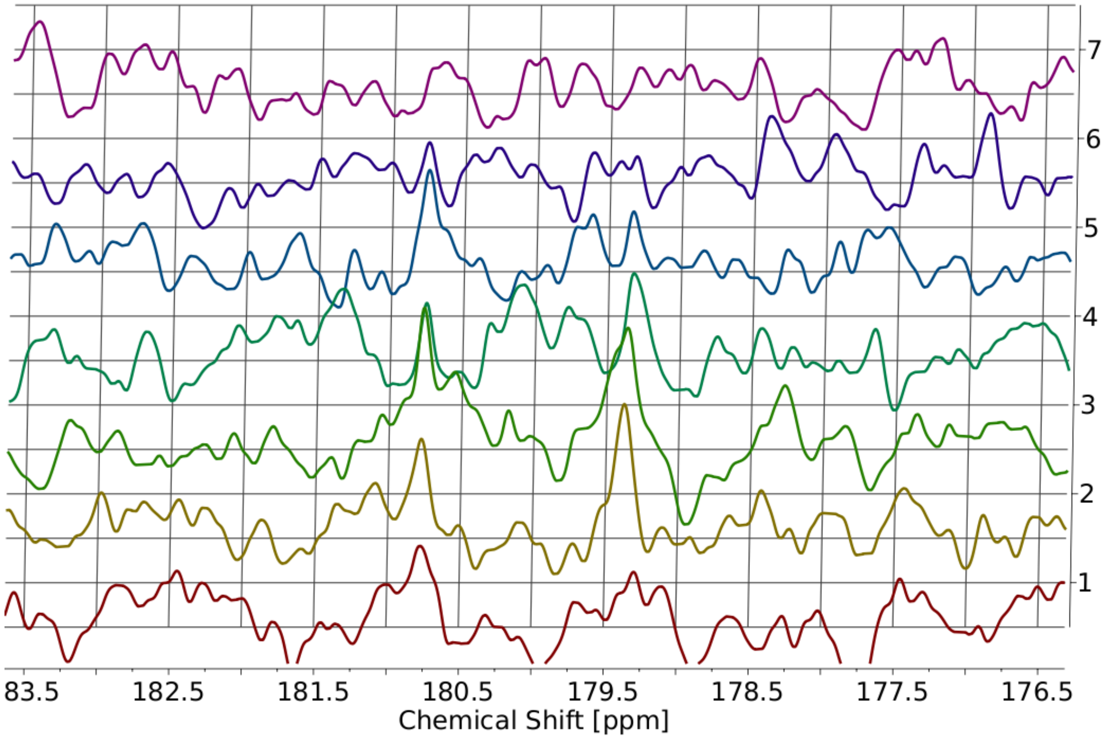

Figure S1. Observing yeasts's metabolism of hyperpolarized fumarate to malate at natural abundance. *A 32 mM hyperpolarized by DNP fumarate solution was injected into the yeast inside the spectrometer to observe the conversion to malate every 4 seconds using a 10° flip-angle. A conversion of fumarate to malate was readily observed, however the signal-to-noise ratio was too low for analysis. Four spectra were summed for each time point shown in this figure.*

Model for metabolic analysis software

Assuming the first-order kinetics in the observed yeast metabolism, we set an ODE system. We expect up to four metabolites (lactate, alanine, CO_2, and bicarbonate in the case of C1 carbon of pyruvate). So, to estimate all T_1 and conversion rates, we created a Python script which fits all data of one substrate at once.

Let's consider first a system containing only the substrate and one product. The rate at which the substrate signal M^S changes will be a function of relaxation R^S and conversion k^S to the productM^P:

$$\frac{dM^S}{dt} = -R^S M^S - k^S M^S$$

Solving this ODE leads to with the boundary condition $M^S(0) = X_0^S$ with X being the name of the molecule and subscript S or P indicating substrate and product. This gives

$$M^S = X_0^S e^{-Ut}$$

With $U = k^S + R^S$. On the other hand, the product will also experience a relaxation R^P as well as the same conversion $k^P = k^S$ in the case of only one product:

$$\frac{dM^P}{dt} = -R^P M^P + k^P M^S = -R^P M^P + k^P X_0^S e^{-Ut}$$

Solving this ODE leads to:

$$M^P = C_2 e^{-R^P t} + \frac{k^P X_0^S}{R^P - U} e^{-Ut}$$

And with $M^P(0) = X_0^P = C_2 + \left(k^P X_0^S\right)/(R^P - U)$ the result is:

$$M^P = \left(X_0^P - \frac{k^P\, X_0^S}{R^P - U}\right) e^{-R^P t} + \frac{k^P\, X_0^S}{R^P - U} e^{-Ut}$$

Keep in mind that the observed relaxation rate $R^{S,P}$ is not only a function of T_1 but also of the excitation angle α and repetition time TR used, which leads to a comprehensive formula for $R^{S,P}$:

$$R^{S,P} = R_1^{S,P} + R_\alpha = \frac{1}{T_1^{S,P}} + \frac{1 - cos(\alpha)}{TR}$$

If we want to describe the kinetics of substrate consumption with multiple products, k^S will be the superposition of all $k_{\text{ges}}^P = k^{P_1} + k^{P_2} + \ldots$ and there will be one more equation for each product M^{P_1}, M^{P_2}, …

The time to the maximum of signal intensity (time2max) can be found analytically for each product. At the $me2max$ $\frac{dM^P}{dt} = 0$, so

$$R^P \left(X^P - \frac{k^P\, X_0^S}{R^P - U}\right) = -\frac{k^P\, X_0^S}{R^P - U} e^{(R^P - U)t}$$

$$t_{max} = \frac{\ln\left(\frac{R^P\left(k^P X_0^S - X_0^P(R^P - U)\right)}{k^P X_0^S U}\right)}{R^P - U}$$

This lets us to find also maximum signal of products $max\%_P = M_P(t_{max})$.

To calculate the AUC we use the integral

$$AUC^P = \int M^P(t)dt = -\frac{k^P X_0^S}{(R^P - U)U} e^{-Ut} - \left(\frac{X_0^P}{R^P} - \frac{k^P X_0^S}{(R^P - U)R^P}\right) e^{-R^P t} + C$$

And if a is the timepoint of the experiment start and b of the end we will have the integral

$$AUC^P = \int_a^b M^P(t)dt$$
$$= \frac{1}{R^P U(U - R^P)}\left(e^{-(U+R^P)a}\left(\left(Ue^{Ua} - R^P e^{R^P a}\right)k^P X_0^S + (U^2 - R^P U)e^{Ua} X_0^P\right)\right.$$
$$\left. - e^{-(U+R^P)b}\left(\left(Ue^{Ub} - R^P e^{R^P b}\right)k^P X_0^S + (U^2 - R^P U)e^{Ub} X_0^P\right)\right)$$

In case we want to standardize the calculation of values, it makes sense to use the individual t_{start} of each metabolite, defined by $M^P(t_{start}) = 0$, and calculate other values based on this timepoint. Since yeast may start metabolizing before the first point is recorded, it is expected that $t_{start} \leq 0s$:

$$M^P(t_{start}) = 0 = \left(X_0^P - \frac{k^P X_0^S}{R^P - U}\right) e^{-R^P t_{start}} + \frac{k^P X_0^S}{R^P - U} e^{-Ut_{start}}$$

$$1 - \frac{X_0^P(R^P - U)}{k^P X_0^S} = e^{(R^P - U)t_{start}}$$

$$t_{start} = \frac{ln\left(1 - \frac{X_0^P}{X_0^S}\frac{R^P - U}{k^P}\right)}{R^P - U}$$

And for normalization purposes with $a = t_{start}$ and $b = inf$ we get

$$AUC_{norm}^P = \int_{t_{start}}^{\infty} M^P(t)dt$$
$$= \frac{1}{R^P U(U - R^P)}\left(e^{-(U+R^P)t_{start}}\left(\left(Ue^{Ut_{start}} - R^P e^{R^P t_{start}}\right)k^P X_0^S\right.\right.$$
$$\left.\left. + (U^2 - R^P U)e^{Ut_{start}} X_0^P\right)\right)$$

We used such a system of ODEs to fit observed metabolic kinetics using Broyden–Fletcher–Goldfarb–Shanno algorithm (minimize function in Python). Boundaries were set to $k^{P\text{x}} \geq 0$, $200s^{-1} \geq R^{S,P} \geq 0.005s^{-1}$ and $1 \geq X_0^{S,P} \geq -1$. This lets us calculate the conversion rate constants $k^{P\text{x}}$ of each product, relaxation rates $R_1^{S,P}$ and starting values $X_0^{S,P}$. We use these values to also

calculate the area under the curves (AUC), time from start to reach the maximum of each product ($time2max$), and the intensity in % of the X_0^S at the $time2max$ ($max\%^P$). Normalized AUC_{norm} was used across all experiments to standardize the results and make them independent of the timepoint of the first recorded spectrum. $time2max$ was also normalized to this t_{start}.

Protocol for the analysis of hyperpolarization spectroscopy

To conform the raw data into metabolic information several steps need to be undertaken.

First, the spectrum needs to be analysed. The 2D acquisition gets imported to MNova, followed by setting of 1 Hz exponential filtering and automated global phase correction (for first and same for subsequent spectra). If the phase is significantly different for the first few spectra, these are discarded due to expected perturbation in the solution which may lead to wrong observations.

The processed spectra are copied for however many substrates and expected products and the region of interest is magnified. Polynomial 3rd-grade baseline correction is applied for the region around the metabolite and the metabolite is integrated. The x-axis is applied according to the repetition time TR used.

All the integral data is saved to an Excel sheet. From this, a CSV file for each substrate and all the corresponding products (e.g. one file for [1-^{13}C]pyruvate and lactate, alanine and BCO_2 plus another file for [2-^{13}C]pyruvate and ethanol) are produced with the first column being the substrate and the following up to four products. Empty columns will be ignored.

Then refer to the CSV file just created in the Python script. Adjust the substrate and product names if necessary and enable or disable certain products by setting "use_X" variable to 1 (yes) or 0 (no). Run the script and check the coefficient of determination R^2 and the plot as quality control. If the fit for certain metabolites is off, use weight_X to change the weighting of the residuals from the fit to improve it. Alter Prod_scale_X to adjust the visual scaling of the product maximum in the plot relative to the substrate (1 is the same height, 0.5 is 50%). If the data is ready to be saved, set writeDataToFile = "yes". A .csv export of the fitted graphs, the corresponding .png plot as well as all the values acquired from the fit as .txt file are saved in the location of the original data csv file. The exported values are ready to be used for further statistical analysis.
